# Highly-Immunogenic Virally-Vectored T-cell Vaccines Cannot Overcome Subversion of the T-cell Response by HCV during Chronic Infection

**DOI:** 10.3390/vaccines4030027

**Published:** 2016-08-02

**Authors:** Leo Swadling, John Halliday, Christabel Kelly, Anthony Brown, Stefania Capone, M. Azim Ansari, David Bonsall, Rachel Richardson, Felicity Hartnell, Jane Collier, Virginia Ammendola, Mariarosaria Del Sorbo, Annette Von Delft, Cinzia Traboni, Adrian V. S. Hill, Stefano Colloca, Alfredo Nicosia, Riccardo Cortese, Paul Klenerman, Antonella Folgori, Eleanor Barnes

**Affiliations:** 1 Nuffield Department of Medicine, University of Oxford, Oxford OX1 3SY, UK; leo.swadling@ndm.ox.ac.uk (L.S.); johnshalliday@gmail.com (J.H.); christabel_k@hotmail.com (C.K.); anthony.brown@ndm.ox.ac.uk (A.B.); ansari.azim@gmail.com (A.A.); david.bonsall@ndm.ox.ac.uk (D.B.); r.richardson@ucl.ac.uk (R.R.); felicity.hartnell@worc.ox.ac.uk (F.H.); annette.vondelft@chch.ox.ac.uk (A.V.D.); adrian.hill@ndm.ox.ac.uk (A.V.S.H.); paul.klenerman@ndm.ox.ac.uk (P.K.); 2 Oxford NIHR BRC, and Translational Gastroenterology Unit, Oxford OX3 9DU, UK; jane.collier@msd.ox.ac.uk; 3 Royal Melbourne Hospital, Parkville, Victoria 3050, Australia; 4 Reithera Srl (former Okairos Srl), Viale Città d’Europa, 679, Rome 00144, Italy; Stefania.Capone@reithera.com (S.C.); Virginia.Ammendola@reithera.com (V.A.); Virginia.Ammendola@reithera.com (M.D.S.); Cinzia.Traboni@reithera.com (C.T.); Stefano.Colloca@reithera.com (S.C.); alfredo.nicosia@reithera.com (A.N.); Antonella.Folgori@reithera.com (A.F.); 5 The Jenner Institute, University of Oxford, Oxford, OX3 7DQ, UK; 6 CEINGE, via Gaetano Salvatore 486, Naples 80145, Italy; 7 Department of Molecular Medicine and Medical Biotechnology, University of Naples Federico II, Via S. Pansini 5, Naples 80131, Italy; 8 Keires AG, Bäumleingasse 18, Basel CH 4051, Switzerland; Riccardo.Cortese@reithera.com

**Keywords:** therapeutic vaccination, adenovirus, modified vaccinia Ankara, immunotherapy, HCV, T-cells, exhaustion

## Abstract

An effective therapeutic vaccine for the treatment of chronic hepatitis C virus (HCV) infection, as an adjunct to newly developed directly-acting antivirals (DAA), or for the prevention of reinfection, would significantly reduce the global burden of disease associated with chronic HCV infection. A recombinant chimpanzee adenoviral (ChAd3) vector and a modified vaccinia Ankara (MVA), encoding the non-structural proteins of HCV (NSmut), used in a heterologous prime/boost regimen induced multi-specific, high-magnitude, durable HCV-specific CD4+ and CD8+ T-cell responses in healthy volunteers, and was more immunogenic than a heterologous Ad regimen. We now assess the immunogenicity of this vaccine regimen in HCV infected patients (including patients with a low viral load suppressed with interferon/ribavirin therapy), determine T-cell cross-reactivity to endogenous virus, and compare immunogenicity with that observed previously in both healthy volunteers and in HCV infected patients vaccinated with the heterologous Ad regimen. Vaccination of HCV infected patients with ChAd3-NSmut/MVA-NSmut was well tolerated. Vaccine-induced HCV-specific T-cell responses were detected in 8/12 patients; however, CD4+ T-cell responses were rarely detected, and the overall magnitude of HCV-specific T-cell responses was markedly reduced when compared to vaccinated healthy volunteers. Furthermore, HCV-specific cells had a distinct partially-functional phenotype (lower expression of activation markers, granzyme B, and TNFα production, weaker in vitro proliferation, and higher Tim3 expression, with comparable Tbet and Eomes expression) compared to healthy volunteers. Robust anti-vector T-cells and antibodies were induced, showing that there is no global defect in immunity. The level of viremia at the time of vaccination did not correlate with the magnitude of the vaccine-induced T-cell response. Full-length, next-generation sequencing of the circulating virus demonstrated that T-cells were only induced by vaccination when there was a sequence mismatch between the autologous virus and the vaccine immunogen. However, these T-cells were not cross-reactive with the endogenous viral variant epitopes. Conversely, when there was complete homology between the immunogen and circulating virus at a given epitope T-cells were not induced. T-cell induction following vaccination had no significant impact on HCV viral load. In vitro T-cell culture experiments identified the presence of T-cells at baseline that could be expanded by vaccination; thus, HCV-specific T-cells may have been expanded from pre-existing low-level memory T-cell populations that had been exposed to HCV antigens during natural infection, explaining the partial T-cell dysfunction. In conclusion, vaccination with ChAd3-NSmut and MVA-NSmut prime/boost, a potent vaccine regimen previously optimized in healthy volunteers was unable to reconstitute HCV-specific T-cell immunity in HCV infected patients. This highlights the major challenge of overcoming T-cell exhaustion in the context of persistent antigen exposure.

## 1. Introduction

Hepatitis C virus (HCV) infection is a leading cause of death and morbidity from liver disease and, with approximately 185 million people infected, represents a significant global health problem [1]. HCV is able to persist in the majority of immune-competent hosts it infects, and this leads to a state of chronic hepatic inflammation, which can progress to fibrosis and cirrhosis of the liver and, ultimately, liver failure or hepatocellular carcinoma.

Despite great advances in the treatment options for HCV [2] there remains a compelling argument for the continued development of not only prophylactic, but also therapeutic, candidate vaccines for HCV [3]. It is likely that, globally, a significant proportion of HCV infected patients will not have access to treatment and surveillance programs [4], as has been the case for antiretroviral therapy for HIV [5], and reinfection is a problem in high-risk cohorts [6,7]. An effective stand-alone therapeutic vaccine or, more likely, a vaccine to be used as an adjunct to IFN-free directly-acting antiviral therapy, to reduce therapy duration and cost, or to prevent persistence on reinfection, remains a desirable global health target.

A T-cell vaccine for HCV is a realistic goal since a significant number of individuals spontaneously clear HCV in the setting of an appropriate immune response, and there is evidence of protective immunological memory against HCV in chimpanzees and humans, where secondary infection is associated with reductions in peak and duration of viraemia, hepatic inflammation, and an increased rate of viral clearance [8,9,10]. Comparative analyses of individuals with distinct clinical outcomes have been performed by several groups, and there is now some consensus on the immune response required to prevent persistence of HCV, but there is no single correlate of protection (reviewed in [11,12]). A strong, broad, and persistent HCV-specific T-cell response during acute infection is required for spontaneous clearance [13,14,15]. T-cell dysfunction is a hallmark of chronic viral infection, including T-cell exhaustion, inhibition by regulatory T-cells, and T-cell escape. Whether a T-cell vaccine can overcome these obstacles, by enhancing existing weak responses or by generating de novo T-cell responses once chronic disease is established is a fundamental question that will be addressed in this study.

Previous immunotherapeutic vaccine approaches [16] have included HCV peptide vaccines [17], recombinant yeast expressing an HCV NS3-core fusion protein [18], autologous dendritic cells loaded with lipopeptides [19], and DNA vaccines [20]. In each case only transient low-level effects were seen on T-cell induction or HCV viral load. More recently repeated vaccination in HCV infected patients with MVA encoding HCV non-structural proteins in combination with pegylated interferon-α and ribavirin (PEG-IFNα/rib) was associated with the induction of HCV-specific T-cells at low levels with a non-significant increase in SVR rates in the vaccinated arm [21,22]. However, studies of HCV immunotherapy have often not evaluated the effect of vaccination in the context of the circulating viral sequence.

We have recently developed a T-cell vaccine for HCV, employing a chimpanzee-derived adenovirus 3 (ChAd3) prime and a human adenovirus 6 (Ad6) encoding the non-structural region of HCV (NSmut; genotype 1b) in a prime-boost regimen. Limitations of this regimen included a modest effect of boost vaccination and poor induction of CD4+ HCV-specific T-cells in healthy volunteers [23], and induction of low magnitude, narrowly-targeted, HCV-specific T-cell response in HCV infected patients [24].

We have since optimised this vaccine regimen in healthy volunteers by employing an MVA-NSmut boost vaccination, which induces high-magnitude polyfunctional CD4+ and CD8+ T-cells, targeting multiple HCV antigens in healthy volunteers [25]. Here, we test whether this optimised regimen is safe and immunogenic in HCV infected patients. An important comparison is made between the immunogencity of this vaccine regimen in healthy volunteers [25] vs. HCV infected patients. We also assess whether the ChAd3-NSmut/MVA-NSmut vaccine regimen can safely induce a more balanced CD4+ and CD8+ T-cell response, of enhanced breadth, magnitude, and functionality compared to a ChAd3-NSmut/Ad6-NSmut vaccine regimen in HCV infected patients, as it does in healthy volunteers.

In a mouse model of persistent viral infection (lymphocytic choriomeningitis virus (LCMV)) T-cell induction by vaccination was reduced in the presence of high viral loads [26,27]. We, therefore, assessed vaccine immunogenicity in patients with either high (untreated), intermediate, or low viral loads (after lead-in of PEG-IFNα/rib before vaccination) to determine the impact of viral load on vaccine immunogenicity. Since HCV may undergo mutation and rapidly escape T-cell immunity, it is essential to assess whether T-cells induced by vaccination effectively recognise a patient’s autologous viral sequence. We use full-length viral sequencing and epitope mapping to evaluate the interaction between T-cell induction and viral sequences at key antigenic targets.

## 2. Materials and Methods

### 2.1. Patient Enrolment and Study Arms

Patients aged 18–65 with HCV genotype-1 were eligible for inclusion. Patients with HIV, HBV, immunosuppressive illness, or evidence of cirrhosis (clinical, biochemical, or histological) were excluded (patient demographics; Table 1). Patients were recruited at Oxford University NHS Trust. The study (EudraCT: 2009-018260-10) was approved by the Medicines and Healthcare products Regulatory Agency (MHRA), and registered in the ClinicalTrial.gov database (ID: NCT01296451). All volunteers gave written informed consent before enrolment, and the studies were conducted according to the principles of the Declaration of Helsinki and in accordance with good clinical practice.

Study arms and vaccination regimes are detailed (Supplementary Materials Table S1). Fifteen volunteers were screened and 13 enrolled into arms A and B, receiving concurrent PEG-IFNα/rib therapy (48 weeks), or arm C, receiving vaccine alone. Patients received 2.5 × 10^10^ viral particles (vp) ChAd3-NSmut prime and MVA-NSmut 2 × 10^8^ PFU eight weeks later. A dose escalation for ChAd3-NSmut is described in [24]. The MVA dose was selected on the basis of the use of MVA vectors in human studies [25,28,29]. One patient (356) received both ChAd3-NSmut and MVA-NSmut vaccinations but withdrew shortly after boost vaccination and, therefore, data from this patient is only included in Table 1. Fourteen weeks after starting PEG-IFNα/rib all patients in arm A had a >2 log drop in viral load before vaccination. Arm B received 2.5 × 10^10^ vp two weeks after starting PEG-IFNα/rib. Vaccines were administered intramuscularly.

ChAd3-NSmut/MVA-NSmut vaccination of healthy volunteers [25] is a separate arm of the same clinical trial as the HCV infected patients described here; this trial is registered as ID: NCT01296451 in the ClinicalTrial.gov database. Comparisons are also made with previously published vaccine studies employing ChAd3-NSmut/Ad6-NSmut in HCV infected patients (HCV002) registered as ID: NCT01094873 [24].

### 2.2. Vaccine Constructs

The chimpanzee adenovirus 3 (ChAd3) vector encoding NS3-5B (1985 amino-acids) of genotype-1b BK strain HCV (accession number M58335) has been described previously [30,31] and was manufactured at the Clinical BioManufacturing Facility, Oxford University. A modified vaccinia Ankara construct containing the same transgene as described above has been previously described [25].

### 2.3. Peptides and Antigens

Four hundred ninety-four peptides, 15 amino-acids long, overlapping by 11 amino-acids (BEI Resources, Manassas, VA, USA), were divided into six pools (F–M) corresponding to NS3p, NS3h, NS4, NS5A, NS5B I, and NS5B II (mean 82, range 73–112 peptides/pool) matching HCV genotype 1B strain BK. For cross-reactivity assays peptides were synthesised by ProImmune, Oxford. PepTivatorAdV5 Hexon pool was supplied by Miltenyi Biotec (Cologne, Germany). Peptides were used at 3 µg/mL and 1 µg/mL for ELISpots and ICS, respectively.

### 2.4. IFNγ-ELISpot Assays

IFNγ-ELISpot assays were performed ex vivo in triplicate at 2 × 10^5^ peripheral blood mononuclear cells (PBMC)/well. PBMC were separated via density gradient and counted using a Guava Personal Cell Analyser (Merck Millipore, Darmstadt, Germany). Internal controls were DMSO and R10 (negative controls) and Concavalin A, FEC (HLA class I-restricted peptides from influenza, Epstein-Barr virus, and CMV), and CMV lysate (Virusys Corporation, Taneytown, MD, USA).

A positive cut off for HCV-specific T-cell responses in the IFNγ-ELISpot assay was defined in both HCV infected patients [24] and healthy volunteers [25] separately using: (i) mean + 3 × standard-deviation of SFC/10^6^ PBMC in negative control wells, giving ≥39 SFC/10^6^ PBMC/pool in HCV infected patients and ≥48 SFC/10^6^ PBMC/pool in healthy volunteers; (ii) and ≥ three times background (mean SFC/10^6^ PBMC in DMSO well); and (iii) positivity in response to vaccination in HCV patients was also defined as a >30% increase in pre-existing responses, or the induction of T-cells targeting new antigens.

Positive pools were summed and the background subtracted. Epitope specificities were determined using frozen PBMC rested overnight, by dividing positive peptide pools into minipools of approximately 10 peptides, then mapping to 15 amino acid long peptides and optimal length peptides. CD8+ T-cell depletion IFNγ-ELISpots were performed using individual peptides and negative depletion beads (Dynal magnets).

### 2.5. Intracellular Cytokine Staining

PBMC at 1 × 10^6^ cells/100 µL were stimulated with peptide pools (F + G + H = NS3/4, I + L + M = NS5A/B) or PMA (phorbol 12-myristate 13-acetate)/ionomycin (50 and 500 ng/mL respectively), or unstimulated (DMSO; 5 ng/mL). Brefeldin-A was added (10 µg/mL) 1 h later, cells incubated overnight (37 °C), stained with fixable-NIR live/dead dye (Life Technologies, Carlsbad, CA, USA), fixed and permeabilised (Foxp3 Fix/Perm kit, BD Biosciences, San Jose, CA, USA), then stained with CD3-Pacific Orange, CD4-Qdot605, CD8-peridinin chlorophyll protein (perCp) Cy5.5, IFNγ-AlexaFluor700 (1:50), IL2-APC (allophycocyanin), TNFα-PE (phycoerythrin)-Cy7, IL17-PE in perm-buffer (30 min room temperature (RT)). All flow cytometry was performed on a BD LSRII machine and analysed using FlowJo v9.5.2 (Treestar, Ashland, OR, USA).

### 2.6. Thymidine Incorporation Proliferation Assay

Thymidine incorporation assays were performed ex vivo as previously described [23], on fresh PBMC, 2 × 10^5^ PBMC/well (in triplicate) with HCV proteins (1 µg/mL; Mikrogen GMBh, Neuried, Germany) and [^3^H] thymidine incorporation. A stimulation index (SI) ≥ 3 was considered positive.

### 2.7. HLA Class I Pentamer Staining

Frozen PBMC were thawed and stained ex vivo with HLA*0201 or HLA*0101 PE-labelled pentamers (Proimmune, Oxford, UK; 20 min in PBS, RT), stained with LIVE/DEAD Fixable Near-IR Dead Cell Stain Kit (Thermo Fisher, Carlsbad, CA, USA; 20 min RT), fixed (1% formaldehyde in PBS, 20min RT), then stained with: CD3-Pacific-Orange, CD8-Pacific-Blue, and either stained with CCR7-PeCy7, CD45RA-FITC, CD38-PerCP5.5; or PD-1-PeCy7, Tim3-AlexaFluor700, CTLA-4-APC, 2B4-PerCpCy5.5 for 30 min RT; or permeabilised (10× permeabilisation buffer; ebiosciences, San Diego, CA, USA) and stained with PD1-PeCy7, Perforin-FITC, Granzyme B-AlexaFluor700 30 min RT.

For intranuclear staining, PBMC were stained as above for pentramers and Live/Dead dye, and then surface stained with: CD3-Pacific-orange, CD8-AlexaFluor700, CD45RA-BV421, CCR7-Pe-Cy7 (30 min RT). PBMC were then fixed (1 h RT) and permeabilised using the Foxp3/Transcription Factor Staining Buffer Set (ebiosciences; 45 min RT) and stained with eomes-eFluor660 and Tbet-BV605.

### 2.8. HCV RNA Quantification

HCV RNA levels (lower limit of quantification 15 IU/mL) and subtype were determined at the virology laboratory, John Radcliffe Hospital, Oxford (COBAS AMPLICOR HCV test v2).

### 2.9. HCV Viral Sequencing

Autologous virus was sequenced at baseline and viral relapse. Thawed plasma (500 µL) was pelleted by centrifugation (23,600× *g* at 4 °C for 60 min) and resuspended in 140 µL of plasma. Viral RNA was extracted using a QIAmp Viral RNA mini kit (Qiagen, Hilden, Germany).

For Sanger sequencing RNA was reverse transcribed and first-round PCR was performed using Superscript III One-Step RT-PCR (Invitrogen, Carlsbad, CA, USA) with specific primers and PCR cycling conditions [24]. Second-round PCR used High Fidelity *Taq* DNA polymerase (Roche, Burgess Hill, UK). PCR products were gel or PCR purified (Qiagen). Products were sequenced bidirectionally using second-round internal primers and Prism Big Dye (Applied Biosystems) on an ABI 3100 automated sequencer. Cycling conditions were: 96 °C × 1 min, followed by 30 cycles of 96 °C × 15 s, 50 °C × 10 s, 60 °C × 4 min. Sequences were analysed and aligned using Sequencher (Version 4.10.1, Gene Codes Corporation, Ann Arbor, MI, USA) and Se-AI (Version 2.0 a11, http://tree.bio.ed.ac.uk/software/).

Libraries were prepared for Illumina full-length viral sequencing using the NEBNext^®^ Ultra™ Directional RNA Library Prep Kit for Illumina^®^ (New England Biolabs, Ipswich, MA, USA) with 5 µL sample (maximum 10 ng total RNA) and previously published modifications of the manufacturer’s guidelines (Version 2.0) [32], briefly: fragmentation for 5 or 12 min at 94 °C, omission of Actinomycin D at first-strand reverse transcription, library amplification for 15–18 PCR cycles using custom indexed primers [33] and post-PCR clean-up with 0.85× volume Ampure XP (Beckman Coulter, High Wycombe, UK).

Libraries were quantified using Quant-iT™ PicoGreen^®^ dsDNA Assay Kit (Invitrogen) and analysed using Agilent TapeStation with a D1K High Sensitivity kit (Agilent, Santa Clara, CA, USA) for equimolar pooling, then re-normalized by qPCR using the KAPA SYBR^®^ FAST qPCR Kit (Kapa Biosystems, Wilmington, MA, USA) for sequencing. Metagenomic virus RNA-Seq libraries were sequenced with 100 base-paired end reads on the Illumina HiSeq 2500 with v3 Rapid chemistry (San Diego, CA, USA).

De-multiplexed sequence read-pairs were trimmed of low-quality bases using QUASR v7.01 [34] and adapter sequences with CutAdapt Version 1.7.1 [35] and subsequently discarded if either read had less than 50b remaining sequence or if both reads matched the human reference sequence using Bowtie Version 2.2.4 [36]. The remaining read pool was screened against a BLASTn database containing all 165 HCV genomes [37] covering its diversity both to choose an appropriate reference and to select those reads which formed a majority population for de novo assembly with Vicuna v1.3 [38] and finishing with V-FAT v1.0 (http://www.broadinstitute.org/scientific-community/science/projects/viral-genomics/v-fat). Reads were mapped back to the assembly using Mosaik v2.2.28 [39], variants were called by V-Phaser v2.0 [40], and intra-host diversity was explored with V-Profiler v1.0 [41].

### 2.10. Sequence Variability at T-cell Epitopes at a Population Level

HCV sequences were downloaded from the Los Alamos database (http://hcv.lanl.gov/content/index). Incomplete sequences and non-genotype 1a or 1b sequences were excluded.

### 2.11. nAbs to ChAd3 Vector

An assay measuring the neutralising antibodies against ChAd3 vector were performed as previously described using recombinant adenoviruses expressing the reporter geneSEAP [42].

### 2.12. Statistical Analysis

Data was assumed to have a non-Gaussian distribution. Non-parametric tests were used throughout; paired tests within an individual (Wilcoxon matched-pairs signed rank test) or unpaired tests between individuals (Mann-Whitney). For correlations, Spearman’s r test was used. Two-tailed *p*-values were used throughout. A *p* value < 0.05 was considered significant. Prism v. 5.0d for Mac was used for analysis. * = *p* ≤ 0.05; ** = *p* ≤ 0.01; *** = *p* ≤ 0.001; **** = *p* < 0.0001.

## 3. Results

### 3.1. Patient Characteristics and Virological Outcome

Patients infected with genotype 1 HCV were vaccinated with ChAd3-NSmut after 14 (arm A) or two (arm B) weeks lead-in of PEG-IFNα/rib treatment, or without treatment (arm C), and all were boosted with MVA-NSmut eight weeks after prime vaccination (Supplementary Materials Table S1).

All vaccinated HCV infected patients were Caucasian, nine of 13 were male, median age 49 (range 35–59) years, mean baseline alanine transaminase (ALT) 67 (range 21–295) IU/mL, and median baseline HCV RNA 1.8 × 10^6^ (range 32,000–33.7 × 10^6^) IU/mL (Table 1). Of the patients treated with PEG-IFNα/rib, 25% achieved sustained virological response (SVR; undetectable HCV RNA six months post-completion of treatment) and one was lost to follow-up. One patient (354) ceased PEG-IFN/rib due to a failure to achieve an early virological response (HCV RNA undetectable after 12 weeks of treatment (EVR) but continued to give blood for immunological assessment. Another patient (356) recommenced intravenous drug use two weeks after receiving boost vaccination and withdrew consent for ongoing trial participation.

### 3.2. Vaccination with ChAd3-NSmut and MVA-NSmut Is Well Tolerated

No serious adverse reactions (ARs) occurred during the course of the study (Supplementary Materials Figure S1A). All localised ARs relating to ChAd3-NSmut vaccination were of mild severity, with local pain occurring in 25% of vaccinees and few systemic ARs being observed, with the most common being myalgia, malaise, and headache in 16.67% of vaccinees.

Local ARs were more common following MVA-NSmut boost vaccination, with local pain occurring in 50% of subjects (median duration 3.4 days; Supplementary Materials Figure S1A). Two patients recorded systemic ARs of moderate severity (syncope and headache), while all other ARs were mild. No significant change in liver biochemistry (ALT) was observed after vaccination. For patient 357, a transient flare in ALT (peak 129 IU/mL) 2.5 months after boost vaccination was attributed to increased alcohol consumption (Supplementary Materials Figure S1B).

### 3.3. Pre-Existing HCV-Specific T-Cell Responses

**Figure 1:**
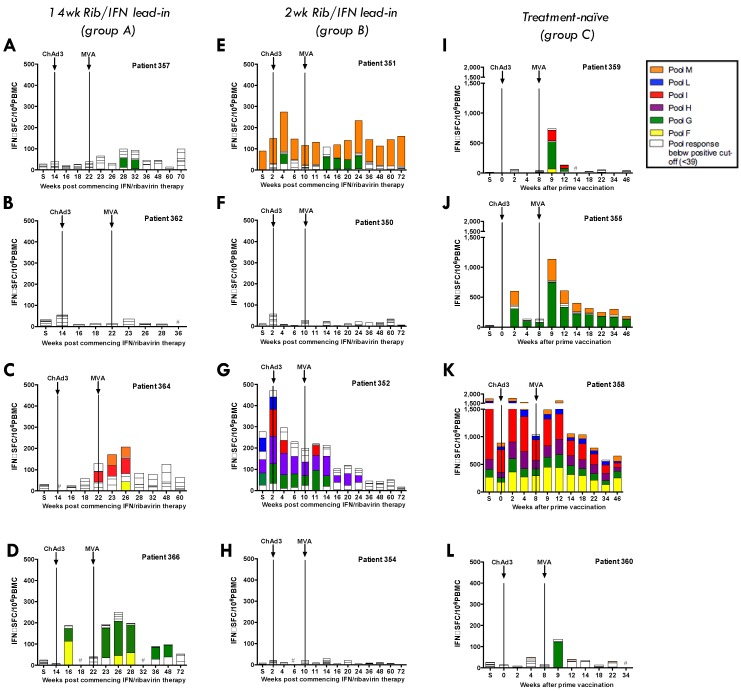
Magnitude of T-cell response targeting HCV NS in ChAd3-NSmut/MVA-NSmut-vaccinated HCV infected patients: the total ex vivo IFNγ ELISpot response to the non-structural region of HCV (NS) encoded in the vaccine, split by peptide pool, is shown across the clinical trial for individual patients after ChAd3-NSmut prime and MVA-NSmut boost vaccination. Positive responses (≥39 SFC/10^6^ peripheral blood mononuclear cells [PBMC]) to a peptide pool are coloured. S = Screen. Vaccinations are indicated by dashed lines. (**A**–**D**) Patients in arm A received 14 weeks of PEG-IFNα/rib treatment (48 weeks total) before vaccination; (**E**–**H**) Patients in arm B received two weeks of PEG-IFNα/rib treatment before vaccination; (**I**–**L**) Patients in arm C are vaccinated, treatment-naïve, HCV infected patients.

Three of the 12 patients enrolled in the trial had detectable pre-existing T-cell responses to the non-structural proteins of HCV (351, 352, 358; Figure 1E,G,K, respectively). Where possible, these responses were mapped to individual 15mer peptides, identifying five epitope targets (Table 2). An epitope prediction programme was used to identify potential optimal sequences within each 15mer (Syfpethi), but these were not tested in vitro due to a lack of available PBMC. Of the five peptides identified, only one contained a previously described epitope (AYAAQGYKVL [43]; Table 2), however, this had been shown to be restricted to HLA-Cw*03, which is not present in patient 358. Another peptide contained the HLA-A2 restricted epitope (QLDLSGWFV), described in a paper investigating the rational design of vaccine immunogens using epitope prediction [44], however, when tested *in vitro*, this optimal peptide did not stimulate a response in patient 351.

### 3.4. Heterologous ChAd3-NSmut Prime/MVA-NSmut Boost Vaccination Can Induce HCV-specific T-cell Responses in Patients Chronically Infected with HCV, But at a Reduced Magnitude When Compared to Healthy Volunteers

**Figure 2:**
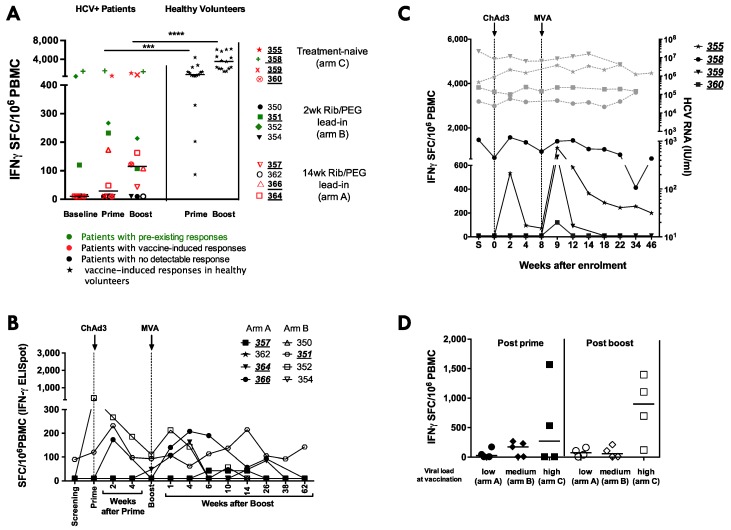
Magnitude of HCV-specific T-cell responses after ChAd3-NSmut (prime) and MVA-NSmut (boost) vaccination in HCV infected patients: the total ex vivo IFNγ ELISpot response to HCV NS is shown (sum of positive pools). Patients with a positive response are underlined. (**A**) Comparison of the total HCV-specific T-cell response in HCV infected patients at baseline and at the peak of the response post-ChAd3-NSmut prime (2–4 weeks post prime) or MVA-NSmut boost (1–6 weeks post boost). Symbols for individual patients are coloured according to whether they had a pre-existing response to HCV NS (green), whether they developed a detectable response post-vaccination (red) or whether no detectable response was seen across the trial (black). The peak T-cell response to HCV NS after ChAd3-NSmut and MVA-NSmut in healthy volunteers (stars) is also shown. Bars at median. Mann- Whitney *t*-test; (**B**) the kinetics of the response in individual HCV infected patients receiving ChAd3-NSmut/MVA-NSmut with two or 14 weeks lead-in of PEG-IFNα/rib pre-vaccination; (**C**) the kinetics of the response in individual HCV infected patients receiving ChAd3-NSmut/MVA-NSmut without PEG-IFNα/rib treatment. HCV RNA also shown (IU/mL; grey dotted lines). S = screening; and (**D**) a comparison of the peak response after ChAd3-NSmut prime and after MVA-NSmut boost vaccination in HCV infected patients that had low (arm A), medium (arm B), or high (arm C) viral loads at the time of vaccination. Bar at median (*p* > 0.05 Kruskal-Wallis multiple comparisons ANOVA for prime, and for boost).

There is no clear pattern to the kinetics of the T-cell response to ChAd3-NSmut/MVA-NSmut vaccination in HCV+ patients (Figure 2A–C), with some patients showing T-cell expansion after: both prime and boost vaccination (patients 351, 366, 358, and 355); after MVA-NSmut boost only (patients 357, 360, and 364) or no novel detectable T-cell response to either vaccine (patients 350, 354, 352, 362). Although the peak magnitude of the T-cell response is seen after MVA-NSmut boost in most patients it varied from one week to six weeks post-vaccination, unlike in healthy volunteers where a peak one week post-MVA-NSmut was consistently seen [25].

When all HCV infected patients are considered together there is no significant induction of HCV-specific T-cells by prime (*p* = 0.2187) or boost vaccination (*p* = 0.2031; Figure 2A); however, when considering responses to individual peptide pools covering the NS region of HCV, four of the eight HCV infected patients receiving PEG-IFNα/Rib treatment (351, 357, 364, 366) had a detectable vaccine-induced T-cell response (a new positive pool or >30% increase in the baseline response to a peptide pool; Figure 2A,B; Table 3). The peak magnitude of the T-cell response is seen after MVA-NSmut boost in most patients (1–6 weeks post vaccination). When compared to the magnitude of the HCV-specific T-cell response induced in healthy volunteers receiving ChAd3-NSmut/MVA-NSmut, the vaccine-induced T-cell responses were significantly lower in magnitude in HCV infected patients (at prime *p* = 0.0003 ***, at boost *p* < 0.0001 ****; Figure 2A).

The relationship between HCV-specific T-cell induction and circulating viral load is best evaluated in arm C, where patients do not have the confounding effect of PEG-IFNα/rib treatment. In this arm 4/4 patients responded to vaccination (Figure 1I–L; 355, 358, 359, 360), developing new or expanded HCV-specific T-cell responses. However, this had no effect on their HCV RNA levels (Figure 2C).

We found no significant difference in T-cell response to vaccination in patients with high (untreated), intermediate (two weeks lead-in of PEG-IFNα/rib), or low (14 weeks lead-in of PEG-IFNα/rib pre-vaccination) viral load at the time of vaccination (Figure 2D). This was unexpected as murine models of chronic viral infection with LCMV have shown that therapeutic vaccination is more efficacious in mice with low viral loads [26,27].

### 3.5. ChAd3/Ad6 vs. ChAd3/MVA Vaccine Regimens in HCV Infected Patients

**Figure 3:**
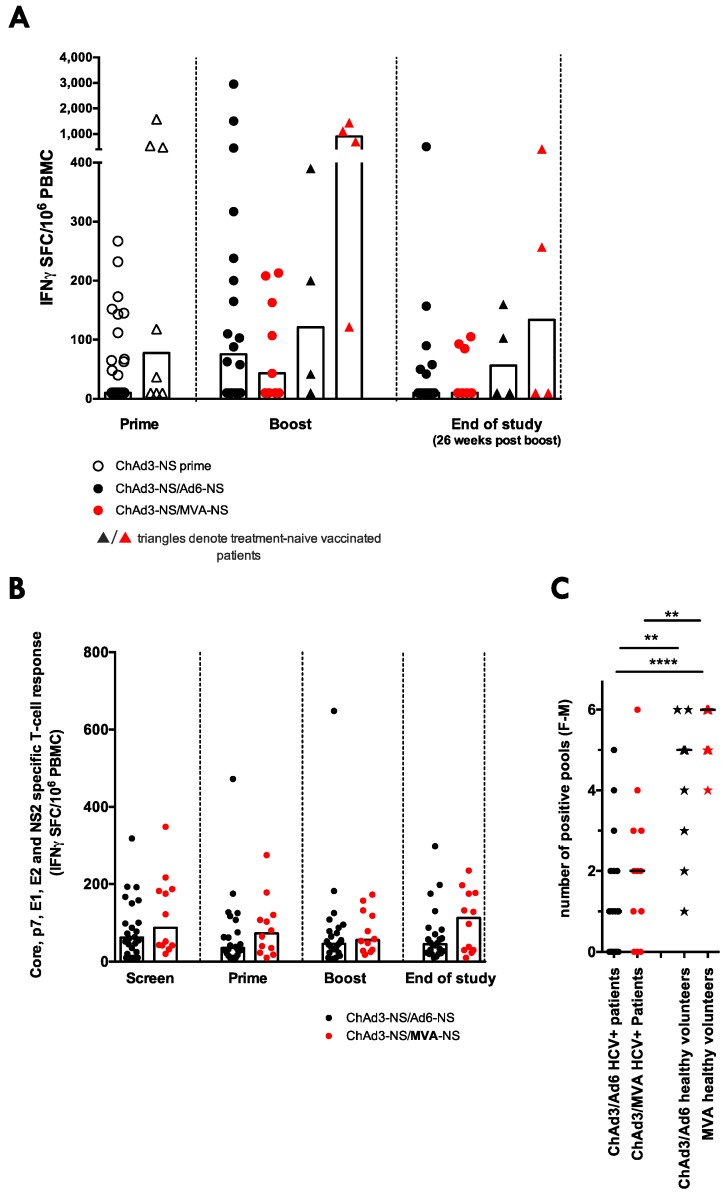
Comparison of the HCV-specific T-cell response in ChAd3-NSmut/MVA-NSmut vs. ChAd3-NSmut/Ad6-NSmut vaccinated HC- infected patients: (**A**) Aa comparison of the peak ex vivo IFNγ ELISpot response to HCV NS (sum of positive pools) in PEG-IFNα/rib treated (circles) or untreated (triangles) HCV infected patients after ChAd3-NSmut prime vaccination (open symbols), and after Ad6-NSmut (black) or MVA-NSmut (red) boost vaccination, and at the end of the study after Ad6-NSmut or MVA-NSmut boost vaccination (Kuskal-wallis unpaired multiple comparisons ANOVA with Dunn’s correction comparing within each time point *p* > 0.05); (**B**) the T-cell response to peptide pools covering the regions of HCV not included in the vaccine immunogen (Core, E1, E2, p7 and NS2; genotype 1b J4 sequence) tested in parallel in patients vaccinated with ChAd3-NSmut/Ad6-NSmut (black) or ChAd3-NSmut/MVA-NSmut (red; Friemand’s paired multiple comparisons ANOVA with Dunn’s correction for ChAd3/Ad6 and for ChAd3/MVA vaccine arms, *p* > 0.05); (**C**) a comparison of the peak breadth of the T-cell response in patients (circles) and healthy volunteers (stars) receiving ChAd3-NSmut/MVA-NSmut (red) or ChAd3-NSmut/Ad6-NSmut (black)—defined as the maximum number of positive pools (out of six pools tested; labelled F M; see Section 2.4) at any time during the vaccine trial. Bar at median. Kuskal-Wallis unpaired multiple comparison ANOVA with Dunn’s correction was applied.

In healthy volunteers MVA-NSmut has shown a greater capacity to boost ChAd3-NSmut primed HCV-specific T-cells, to broaden the T-cell response, and to improve the functionality of the induced HCV-specific T-cells when compared to Ad6-NSmut as a boosting vector [25]. Next we compare the immunogenicity of MVA-NSmut vs. Ad6-NSmut as boosting vectors in HCV infected patients. There was no significant difference in magnitude of the peak (any time after prime vaccination) or memory (measured at the end of the study, 26 weeks after boost vaccination) HCV NS-specific T-cell response in HCV infected patients who received Ad6-NSmut vs. MVA-NSmut boost vaccination, regardless of whether patients received concomitant PEG-IFN-α/rib treatment (Figure 3A). PEG-IFN/rib treatment and concomitant reduction in HCV RNA in itself does not appear to enhanced T-cell responses, however, as only T-cell responses targeting regions of HCV contained within the vaccine immunogen (non-structural region NS3-NS5b) were expanded post-vaccination and the T-cell response targeting the structural regions (Core, E1, E2, p7 and NS2) were unchanged throughout the trial in all patients (Figure 3B; [45]).

The number of positive peptide pools at the peak of the T-cell response was significantly reduced in HCV infected patients relative to healthy controls, and patients who received MVA-NSmut did not have a broader response than those who received Ad6-NSmut boost vaccination (*p* = 0.182; Figure 3C). Therefore, the use of an highly immunogenic vaccine regimen, optimised in healthy volunteers, failed to reconstitute a strong HCV-specific T-cell response in HCV infected patients.

### 3.6. The Relationship between Vaccine Induced T-cell Specificity and Endogenous Viral Sequence

**Figure 4:**
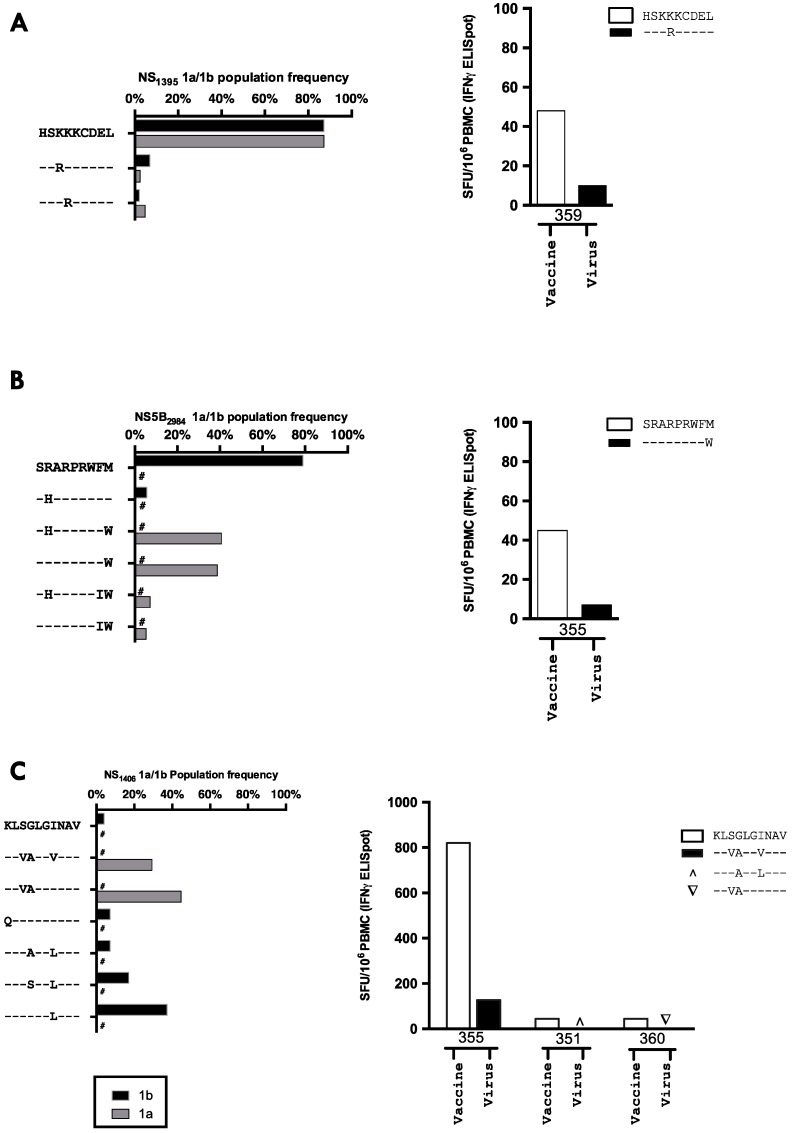
Viral variability at a population level and cross-recognition at HCV T-cell epitopes: (**A**–**C**) left panel The degree of variability in genotype 1 HCV sequences at identified epitopes (grouped by subtype 1a or 1b) is defined using a median of 815 sequences (range 480–839) for each epitope derived from the Los Alamos database. Variants with a global prevalence of >5% of subtype 1a or 1b are shown. The vaccine sequence is given in full and amino acids identical to this sequence are depicted as a – for variant sequences below. # denotes 0% of that subtype had given sequence. The number of available sequences for each epitope varied. (**A**) NS3_1395_ 1a *n* = 839, 1b *n* = 821; (**B**) NS5B_2984_ 1a *n* = 563, 1b *n* = 480; and (**C**) NS3_1406_ 1a *n* = 839, 1b *n* = 809. (**A**–**C**) the right panel shows the ex vivo IFNγ ELISpot response in HCV infected patients vaccinated with ChAd3-NSmut/MVA-NSmut when stimulated with the vaccine sequence peptide (white bars) or with a patient’s autologous viral sequence peptide (black, or symbols if undetectable by ELISpot).

Where possible vaccine-induced T-cell responses to HCV peptide pools were mapped to individual 15mers and optimal peptides; three optimal epitope sequences were identified, all of which had been previously identified in natural infection [44,46,47] or in vaccination in healthy volunteers [23,25] (Figure 4). No CD4+ T-cell responses were identified by peptide mapping.

The viral sequence variability at key epitopes targeted by vaccine-induced HCV-specific T-cells was assessed using the Los Alamos database of HCV sequencing data. The relative frequency of viral variants for genotype 1a and 1b HCV sequences at the epitopes NS3_1406_ (KLSGLGINAV), NS5B_2984_ (SRARPRWFM), and NS3_1395_ (HSKKKCDEL) are shown in Figure 4A–C. The most immunogenic epitope (5/6 HLA-A2+ patients showing a response to this epitope), NS3_1406_, was highly variable with no single epitope being present in more than 50% of genotype 1a and 1b sequences. However, NS3_1395_ was highly conserved, with 87.3% of all natural viral sequences (genotype 1a and 1b combined) and the vaccine sequence being identical.

One explanation for why the induction of HCV-specific T-cells in untreated patients (without the confounding immunomodulatory effects of PEG-IFN-α/rib) had no effect on HCV RNA is a lack of cross-recognition of viral peptides presented on infected cells. We, therefore, performed full-length viral sequencing and assessed the circulating viral sequence at key epitope targets. We confirmed the clinical viral subtyping by producing a phylogenetic tree including patients’ sequences and reference sequencing for HCV subtypes 1a, 1b, 2a, and 3a (Supplementary Materials Figure S2). Clustering of individual patients’ viral sequences when measured at two different time points confirmed the accuracy of full-length viral sequencing.

In all instances where a T-cell response was detected after vaccination the patient’s autologous virus had between one and three amino acid substitutions within the epitope from the vaccine immunogen sequence (Table 4). For instance, the viral sequence in the only patient to make a T-cell response to the HLA-B8 restricted NS3_1395_ epitope was a rare variant (2.1% of sequences in the los Alamos database) HSK**R**KCDEL; in all other patients the viral sequence was identical to the vaccine immunogen, including two HLA-B8+ patients who had no detectable T-cell response to this epitope. The HSK**R**KCDEL variant sequence was poorly recognised by ChAd3-NSmut/Ad6-NSmut induced T-cells (Figure 4A). A vaccine induced T-cell response to NS3_1406_ was detected in five of the six HLA-A2+ patients, all of which had circulating virus with 2 or more amino acids that differed from the vaccine sequence at this epitope. Importantly, when we tested the recognition of peptides corresponding to the patient viral sequence there was limited or no cross-recognition by vaccine-induced T-cells (Figure 4A–C). No change in viral sequence accompanied an induction of HCV-specific T-cells after vaccination at these three antigenic targets (Table 4), which again suggests vaccination is priming or boosting T-cells which have already been escaped by a patients autologous virus. HCV-specific T-cells were only induced by vaccination when there was mismatch between the patient virus and the vaccine immunogen and T-cell induction did not lead to a change in the dominant viral sequence at epitope targets or reduce viral load.

A global defect in T-cell induction was not seen in HCV infected patients as T-cells targeting the hexon of ChAd3 were expanded in all patients (Supplementary Materials Figure S3A). The limited immunogenicity of ChAd3-NSmut in HCV infected patients relative to healthy volunteers cannot be explained by a higher baseline titer of pre-existing neutralising antibodies to ChAd3 as baseline titers were comparable between patients and volunteers (Supplementary Materials Figure S3B). The induction of anti-ChAd3 nAb was also comparable, showing there is also no global defect in the B-cell compartment in patients chronically infected with HCV (Supplementary Materials Figure S3B).

### 3.7. Vaccine-Induced T-Cell Function and Phenotype in Patients with Chronic HCV

**Figure 5:**
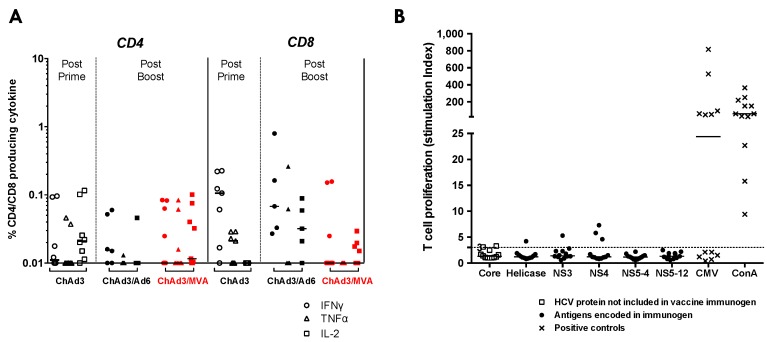
Functional analysis of vaccine-induced HCV-specific T-cells in HCV infected patients: (**A**) cytokine production by PBMC stimulated with peptide pools covering NS3-NS5b. The percentage of total CD4+ or CD8+ cells making IFNγ, TNFα, or IL-2 at the peak post-prime vaccination (2–8 weeks post-ChAd3-NSmut; open symbols), post-Ad6-NSmut boost (2–4 weeks post-Ad6-NSmut; black), or post-MVA-NSmut boost vaccination in HCV infected patients (1 week post-MVA-NSmut red). ICS was performed on patients with an IFNγ ELISpot response >150 SFC/10^6^ PBMC where PBMC were available (ChAd3-NSmut/Ad6-NSmut vaccinees: 024 and 027, and 039; 103 post boost only; ChAd3-NSmut/MVA-NSmut vaccinees: 355, 358, 351, and 352; 360 post-boost only). All values are after background subtraction (DMSO wells). Bars at median; and (**B**) proliferative capacity: the proliferative response to recombinant HCV proteins in patients six weeks post-MVA-NS boost, plotted as Stimulation Index (SI). SI ≥ 3 was considered positive. Responses to positive control antigens (concanavilin A and CMV lysate) are shown (crosses). Bars represent median values.

Next, we used intracellular cytokine staining (ICS) and the thymidine proliferation assays to confirm the limited induction of functional HCV-specific T-cells seen by IFNγ ELISpot assay, to assess specifically, CD4+ T-cell induction and the presence of T-cells with the capacity to proliferate or produce IL-2 and TNFα. Cytokine producing HCV-specific T-cell populations were low in magnitude and MVA-NSmut failed to boost a stronger CD4+ or CD8+ T-cell response relative to Ad6-NSmut boost (Figure 5A). This is in stark contrast to what was seen in healthy volunteers, where MVA-NSmut boost vaccination induced a significantly higher magnitude population of HCV-specific cytokine-producing T-cells, in particular CD4+ T-cells [25]. MVA-NSmut boost vaccination of HCV infected patients failed to induce HCV-specific T-cells that proliferate on stimulation with recombinant HCV proteins (Figure 5B; data shown for stimulation of PBMC six weeks post MVA-NSmut boost; PBMC taken 24–26 weeks post (*n* = 3) and 40–62 weeks post (*n* = 7) MVA-NSmut also showed a stimulation index <7 for all proteins in all patients tested).

**Figure 6:**
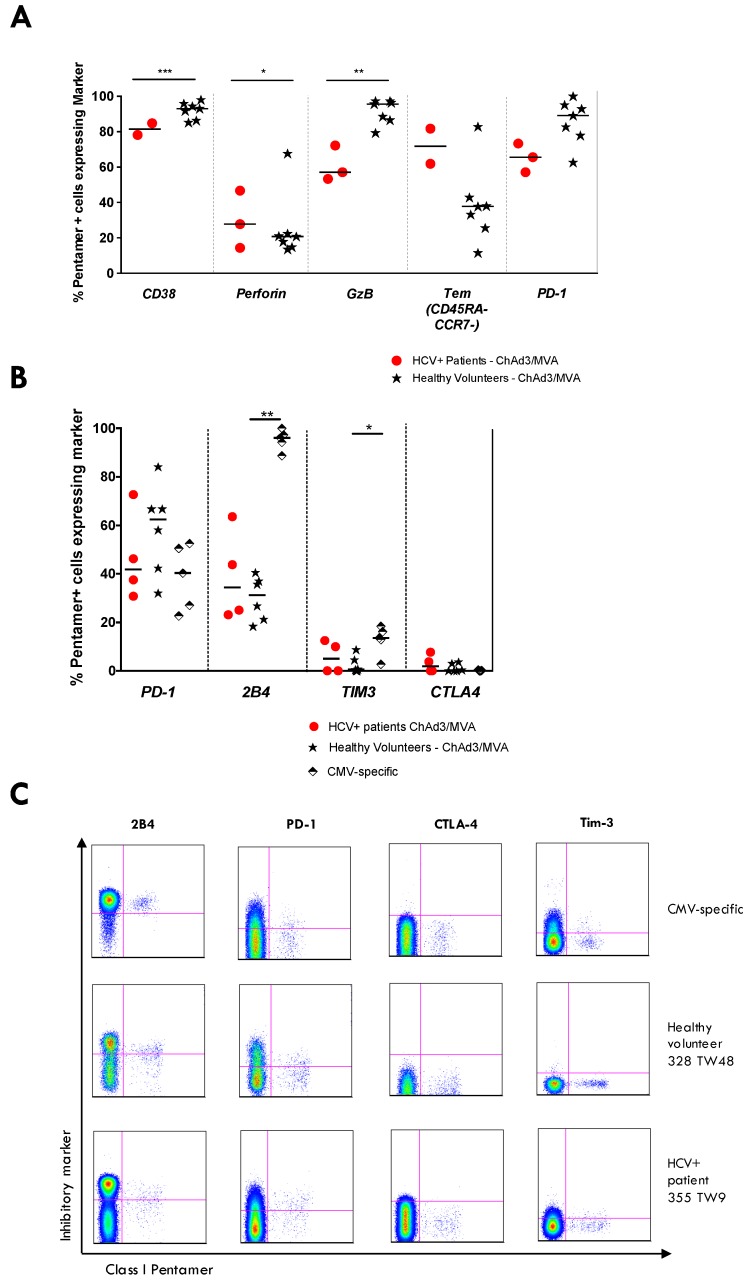
Phenotype of vaccine-induced HCV-specific T-cells in patients vs. healthy volunteers: (**A**–**C**) the percentage of pentamer+ cells (NS3_1436_ ATDALMTY, NS3_1406_ KLSGLGINAV) expressing a given marker in HCV infected patients (red dots; 355, 357, 360) or healthy volunteers (black stars) receiving ChAd3-NSmut/MVA-NSmut prime-boost vaccination. CD8+ CMV-specific T-cells (pp65_495–504_) in healthy volunteers were also assessed. (**A**) Pentamer+ cells were stained ex vivo at the time of peak magnitude of the T-cell response during the trial (as measured by IFNγ ELISpot; 2–4 weeks post ChAd3-NSmut; 1–6 weeks post MVA-NSmut) in HCV infected patients and healthy volunteers. Bars represent median values. Kruskal-Wallis multiple comparison ANOVA with Dunn’s correction and significant differences are illustrated; (**B**) inhibitory receptor staining of pentamer+ cells was performed at the end of the trial (20–62 weeks post MVA-NSmut prime) in HCV infected patients (red dots) and healthy volunteers (black stars), or in CMV+ healthy volunteers (pp65_495–504_ NLVPMVATV); and (**C**) example FACS plots of T-cell surface inhibitory marker staining, gated on total CD8+ T-cells.

The phenotype of HCV NS-specific T-cells at the peak of the response post MVA-NSmut boost was assessed in patients 355, 360, and 357 to investigate whether mismatched T-cells induced by vaccination in HCV infected patients are phenotypically similar to those induced in healthy volunteers. A subset of inhibitory coreceptors characteristic of exhausted HCV-specific T-cells (PD-1, Tim-3, CTLA-4 and 2B4 [48]) were also assessed on vaccine-induced T-cells. HCV NSmut-specific T-cells expressed CD38 and perforin (Figure 6A) immediately after MVA-NSmut vaccination, similar to that seen in healthy volunteers; in all patients fewer pentamer+ cells expressed granzyme B and PD-1 after MVA-NSmut boost in HCV infected patients than in healthy controls. Pentamer+ T-cells in HCV infected patients did not express higher levels of inhibitory receptors than vaccine-induced HCV-specific T-cells in healthy volunteers long-term after vaccination (Figure 6B; example plots shown in Figure 6C).

Vaccine-induced CD8+ T-cells, show a partially dysfunctional phenotype when compared to healthy volunteers, despite not cross-recognising a patient’s autologous viral sequence; in patients, responses are skewed towards a Tem phenotype, but they lack the ability to produce antiviral cytokines, to proliferate when exposed to cognate antigen, and often do not express the molecules indicative of good cytolytic capacity.

**Figure 7:**
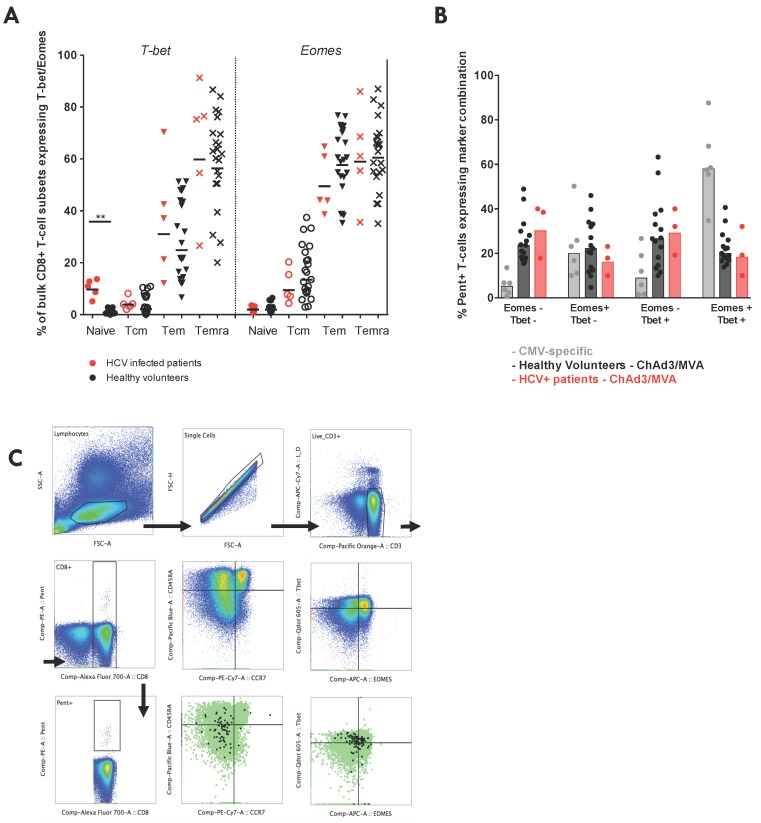
Transcription factor expression by bulk CD8+ T-cells and by vaccine-induced HCV-specific T-cells in patients vs. healthy volunteers: (**A**) the intranuclear expression of the transcription factors Tbet (T-box transcription factor TBX21) and eEomes (eomesodermin) on T-cell memory subsets of bulk CD8+ T-cells (CD45RA+CCR7+, Tn; CD45RA−CCR7+, Tcm; CD45RA−CCR7−, Tem; CD45RA+CCR7−, Temra) in HCV infected patients (red) and healthy volunteers (black). Bars represent median values; (**B**) the percentage of pentamer+ cells (NS3_1406_ KLSGLGINAV) co-expressing a given combination of eomes and Tbet ex vivo in HCV infected patients (red; 355, 357, 366) or healthy volunteers (black stars) at the end of the study after receiving ChAd3-NSmut/MVA-Nsmut prime-boost vaccination (8–44 weeks post-MVA vaccination). CD8+ CMV-specific T-cells (pp65_495–504_) in healthy volunteers were also assessed. Bars represent median values. Kruskal-Wallis multiple comparison ANOVA with Dunn’s correction and significant differences are illustrated; and (**C**) example gating strategy for intranuclear staining.

To investigate whether the partially dysfunctional phenotype of vaccine-induced HCV-specific T-cells is controlled at the level of transcription, we compared the expression of two transcription factors (TFs) that control CD8+ T-cell memory development and effector functions (Tbet, T-box transcription factor TBX21; eomes, eomesodermin) [49]. Bulk CD8+ T-cell memory subsets (CD45RA+CCR7+, Naïve, Tn; CD45RA−CCR7+, Tcm; CD45RA−CCR7−, Tem; CD45RA+CCR7−, Temra) showed distinct TF expression, with Tbet and eomes expression increasing progressively as one moves from the Naïve-Tcm-Tem-Temra phenotype, both in HCV infected patients and healthy volunteers (Figure 7A) [49]. Interestingly, Naïve CD8+ T-cells in HCV infected patients expressed higher levels of Tbet, potentially indicating a bystander activation of this subset during chronic HCV infection, as has been previously described [50]. The vaccine-induced HCV-specific memory T-cells that persisted long-term after vaccination in HCV infected patients showed the characteristic Tbet/eomes coexpression pattern seen by T-cells elicited by ChAd3/MVA prime-boost vaccination in healthy volunteers, which closely mirrored the coexpression seen by CMV-specific T-cells (Figure 7B,C). Despite having reduced effector function, vaccine-induced T-cells in HCV infected patients showed no difference in expression of exhaustion marker or key TFs.

### 3.8. Culturing Pre-Vaccination HCV-Specific T-cells

**Figure 8:**
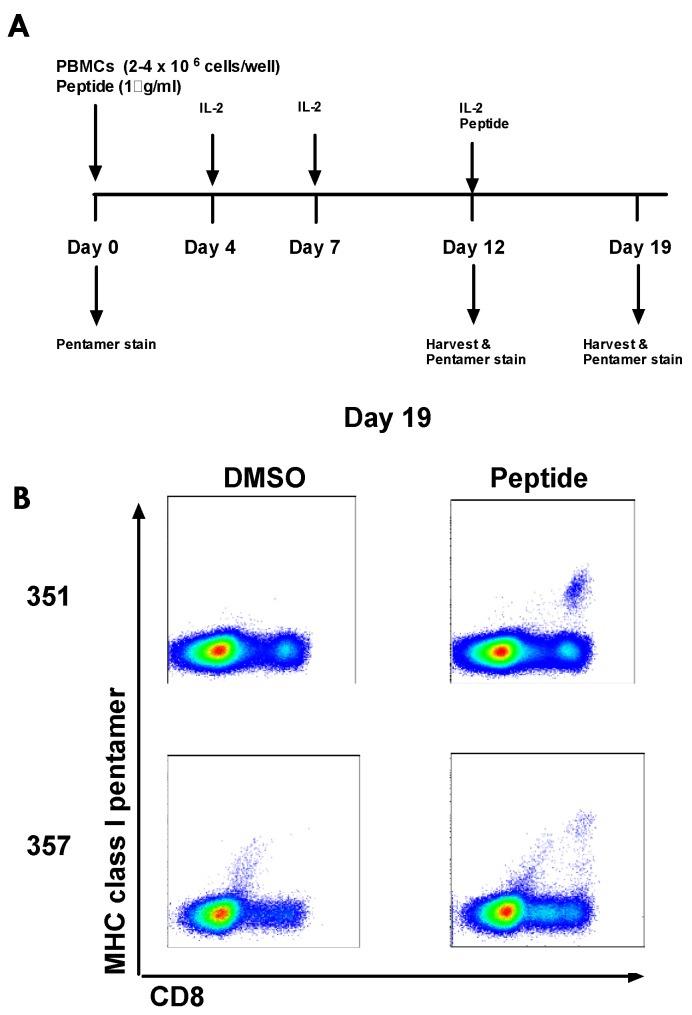
HCV peptide-specific T-cell lines grown from PBMC frozen pre-vaccination in HCV infected patients: (**A**) PBMC frozen pre-vaccination from HLA-A2+ patients who showed a positive response to NS3_1406_ after vaccination were cultured with the 15mer (LAA**KLSGLGINAV**AY; optimal epitope highlighted in bold) peptide and recombinant human IL-2 for 19 days. PBMC were stained with MHC class I pentamer (NS3_1406_ KLSGLGINAV) at day 0, 12, and 19 of the culture; and (**B**) example plots for the two patients for which NS3_1406_-specific T-cells were grown to detectable levels at day 19 from pre-vaccine PBMC (patients 351, 357. Negative at day 19: patients 355 and 360).

One explanation for why vaccine-induced T-cells are not expanded to the same degree as in healthy volunteers and why they are phenotypically different could be that HCV NS-specific T-cells have already been primed during HCV infection in patients and that vaccination is boosting pre-existing, exhausted T-cells rather than priming de novo responses. We identified 4 HLA-A2+ patients who developed a detectable T-cell response after vaccination to NS3_1406_ (but who showed no detectable response pre-vaccination by ICS, pentamer or IFNγ ELISpot; 351, 355, 357, 360) and attempted to expand T-cells to detectable levels from frozen pre-vaccination PBMC. Two of the four HCV infected patients developed pentamer clouds to this epitope by day 19 of culture (all were negative at day 0, day 12, and at all time points when cultured with DMSO; Figure 8). PBMC frozen pre-vaccination from three healthy HLA-A2+ volunteers who responded to this epitope post-vaccination were also cultured; as expected these volunteers did not develop pentamer clouds, showing our cell culture method does not prime de novo responses [51].

In some instances the frequency of antigen-experienced HCV-specific T-cells circulating in HCV infected patients before vaccination may be below the sensitivity limit of IFNγ ELISpot assay; therefore, vaccination may be boosting pre-existing T-cells rather than inducing de novo responses. The absence of cultured responses in all individuals who respond to vaccination could suggest some responses are indeed primed by vaccination, however, it is also possible that our culture method is not sensitive enough to expand all pre-primed responses from a small sample of pre-vaccination PBMC.

## 4. Discussion

It is well established that, in chronic HCV infection, T-cells are found at a low magnitude and are functionally attenuated, with reduced capacity to proliferate and to perform antiviral effector functions. We sought to assess whether a potent T-cell vaccine could overcome these factors, either by enhancing existing weak responses or by generating new HCV-specific T-cell responses. We have recently shown that HCV-specific T-cells can be optimally induced in healthy volunteers by a prime-boost vaccine regimen using a ChAd3-NSmut prime and a MVA-NSmut boost vaccination, overcoming the limitations of previous heterologous ChAd3-NSmut/Ad6-NSmut regimen [25]. Here we assessed the capacity of this potent regimen to restore anti-viral T-cell responses in patients with chronic HCV infection and analysed in detail the complex interplay between HCV-specific T-cell induction and circulating viral variants in the context of T-cell immunotherapy.

Vaccination was well tolerated with no clear evidence of liver immunopathology. Detectable HCV-specific T-cells responses are induced by ChAd3-NSmut/MVA-NSmut vaccination in a subset of patients (4/8 PEG-IFN-α/rib treated and 4/4 untreated patients). However, an important comparison with vaccination in healthy volunteers should be made: HCV-specific T-cell induction by vaccination occurs in some, but not all HCV infected patients, whereas vaccination induces HCV-specific T-cells in all healthy volunteers. Both the magnitude and the breadth of the HCV-specific T-cell response after vaccination are significantly lower in HCV infected patients than healthy volunteers. Although MVA-NSmut is more immunogenic as a boosting vector than Ad6-NSmut in healthy volunteers, in HCV infected patients MVA-NSmut was no more immunogenic than Ad6-NSmut. MVA-NSmut boost vaccination also does not broaden the T-cell response or induce a significant population of HCV-specific CD4+ T-cells in HCV infected patients. In one untreated patient (355) a T-cell response of comparable magnitude and kinetics with that seen in healthy volunteers was observed, however, the response was narrowly targeted, dominated by monofunctional IFNγ CD8+ T-cells and had no effect on HCV RNA. The potency of our T-cell vaccines in healthy volunteers did not predict their immunogenicity in a therapeutic setting.

Despite induction of HCV-specific T-cells in all patients not receiving PEG-IFN/rib, no change in circulating viral load was observed, nor was any change driven by these T-cells in the dominant viral sequence at the epitopes they targeted. Where T-cells were induced, they appeared partially dysfunctional. In order to better understand why some patients responded to vaccination and others did not, the sequence of endogenous virus in patients was evaluated. In vaccine responders there was always sequence divergence between the circulating virus and vaccine immunogen at the epitope targeted by vaccine-induced T-cells and, importantly, there was little or no cross-recognition of the viral sequence by vaccine-induced T-cells in in vitro assays. Often, both responders and non-responders shared the same variant sequence at an epitope pre-vaccination and no viral sequence was predictive of a response to vaccination. Overall, HCV T-cell vaccines may induce T-cell responses in chronic infection, but primarily in the absence of homologous antigen stimulation. The consequence of this is that these T-cells are unlikely to limit viral replication or mediate viral eradication as they will not recognise presented viral peptides on infected hepatocytes or antigen presenting cells. The finding that vaccination had no effect on HCV viral load is in keeping with this. T-cells were not induced when there was sequence homology between the vaccine immunogen and autologous virus at known T-cell epitopes; in this context vaccination was unable to restore functional immunity.

Failure to respond to an epitope where the virus and vaccine have the same sequence may be a result of T-cell exhaustion. Detection of T-cell responses where sequence mismatch occurred may be the result of de novo priming, or expansion of memory responses. In a recent study on chimpanzees, where DAA treatment was combined with therapeutic vaccination (Ad6/ChAd63/DNA or Ad6/MVA/MVA/ChAd63 all encoding NSmut), vaccination failed to expand detectable CD4+ T-cell responses and despite initial viral control DAA-resistant HCV variants emerged, but were not controlled by HCV-specific T-cells [52]. Most vaccine-induced T-cells targeted epitopes that were not conserved in the circulating virus, and where the viral sequence was the same as the vaccine immunogen and remained intact, intrahepatic T-cells were exhausted and failed to control the virus [52]. A sustained multifunctional T-cell response against an intact epitope was observed in the blood, however, localised exhaustion of T-cells targeting this epitope was seen in the liver [52]; therefore, even in the face of an un-escaped T-cell response, rapid exhaustion of T-cells by relatively low level HCV replication and the tolerogenic liver environment can lead to the development of chronic HCV infection.

We found no correlation between HCV viral load at the time of vaccination and the T-cell response to vaccination. This was unexpected as evidence from murine models of chronic viral infection suggest T-cell induction can be limited in the presence of high viral titers [26,27]. However, this may be explained by the observation that PEG-IFNα and ribavirin have been shown to attenuate cellular immune responses [45,53], or by the relatively small sample size. Of note, we found that T-cells targeting the hexon of the Ad vectors were readily expanded on vaccination in patients receiving PEG-IFNα, suggesting that therapy did not induce a global impairment of T-cell durability or expansion. HCV infected patients showed a comparable nAb induction and seroconversion for anti-ChAd3 nAbs, showing that there is no global defect in the B-cell compartment in patients chronically infected with HCV. The recent licensing of IFN-free DAA regimens mean that the immunomodulatory effects of PEG-IFNα/rib will no longer be an issue when testing therapeutic vaccines as an adjunct to DAA therapy.

T-cells generated by vaccination were functionally impaired in HCV infected patients compared to healthy volunteers; they did not proliferate on stimulation with a cognate antigen, few produced antiviral cytokines, and they often lacked markers of cytotoxic capacity, a phenotype that is characteristic of chronic viral exposure [54,55]. A possible explanation for the partial dysfunction of vaccine-induced T-cells is that HCV infection, per se, inhibits the effective priming of naive T-cells to produce functional T-cell memory, however, here, patients generated robust anti-vector T-cell responses. An alternative explanation is that vaccination is not priming truly naive T-cells, but is stimulating memory responses that were generated early in infection. Historic infection with a virus whose sequence matched the vaccine immunogen or the transient emergence of minor viral variants within the swarm of quasi-species within a host that match the vaccine immunogen could prime T-cells that no longer recognise circulating virus but that respond suboptimally to vaccination. The degree of dysfunction could depend on the duration of antigen exposure at the epitope level—with more functional responses associated with viral escape early in infection. In support of this, we found pre-existing T-cell responses below the limit of detection that expanded on vaccination but failed to recognise autologous viral sequences. These partially-dysfunctional vaccine-induced responses did not however express significantly higher levels of inhibitory receptors (PD-1, 2B4, CTLA-4, Tim3) and were not enriched for CD45RA re-expressing Temra phenotype T-cells, despite showing very little proliferative capacity and polyfunctionality.

The transcription factor Tbet controls a programme of gene expression that confers cytolytic potential and production of antiviral cytokines (in particular IFNγ) in CD8+ T-cells [56,57]. Another TF with a somewhat overlapping role in controlling effector functions of T-cells, eomes appears to be required for efficient memory development [58] but has also been associated with T-cell exhaustion [56,59]. It may have been expected that the vaccine-induced HCV-specific T-cells would express a lower level of Tbet and higher eomes expression, indicative of their partially dysfunctional state, as has been shown for virus-specific T-cells in the setting of chronic viral infection [59,60]; however, no difference in the expression of TFs was seen between the vaccine-induced T-cells in HCV infected patients and healthy volunteers, suggesting that these markers are not always indicative of the ability of a T-cell to perform effector functions and alone cannot substitute the direct measurement of cytokine production, proliferative and cytolytic capacity.

A lack of expansion of CD4+ T-cells by vaccination, even where there was sequence mismatch between the immunogen and circulating virus, could indicate that rather than viral escape, other mechanisms of immune evasion are used by HCV to avoid CD4+ T-cell recognition. In one study 75% of CD8+ but only 18% of CD4+ T-cell epitopes showed amino acid changes during the development of persistent HCV infection [61]. However, viral escape of the dominant CD4+ T-cell responses to DNA/rVV vaccination in chimpanzees resulted in persistence of HCV after challenge [62].

It appears that delivery of an antigen to the periphery, away from the tolerogenic liver, in the context of a highly-immunogenic non-replicative viral vectors is not enough to rejuvenate T-cells that effectively recognise circulating HCV in chronically-infected patients. The targeting of co-stimulatory [63,64] or inhibitory pathways [65] may be required to allow T-cells to respond to vaccination and to re-acquire effector functions. In 2/3 HCV chronically-infected chimpanzees PD-1 blockade had no effect on viral load or T-cell responses [66]. One chimpanzee, who had a broader T-cell response during initial infection, did respond to PD-1 blockade, with expansion and broadening of both CD4+ and CD8+ T-cell responses coinciding with a significant drop in viral load, which rebounded on withdrawal of the blockade. The continued assessment of inhibitory receptor blockade is needed as it was shown to be ineffective for severely-exhausted T-cells in the liver in one study [67] and may require concurrent depletion of Tregs to allow anti-viral T-cell expansion rather than selective expansion of Tregs [68].

A limitation of first in human Phase I clinical trials is the small number of study participants (4–5 per arm). The group sizes in this study are typical of those in a phase I study that must balance the requirement of meeting the study end-points (safety and immunogenicity) with ethical constraints. In spite of the small sample sizes, statistically significant differences in immune measurements were found when comparing vaccinated healthy volunteers and HCV infected patients. To fully assess the efficacy of this vaccine regimen and the cross-reactivity of vaccine-induced T-cells across a broader range of HLAs and HCV genotypes, a larger cohort of patients is required. A group of healthy volunteers were vaccinated with the same regimen (ChAd3-NSmut/MVA-NSmut) as part of this study, allowing an accurate comparison to be made with the vaccines immunogenicity in HCV infected patients; however, comparison are also made with historic data from a previous study employing ChAd3-NSmut/Ad6-NSmut in a heterologous prime-boost vaccine regimen [24]. This is a limitation to the study, although both trials were performed in the same laboratories, by the same staff, and using the same experimental setup and reagents, therefore, the relative immunogenicity of these regimens can be reasonably compared in HCV infected patients.

This work highlights the major challenges in developing an immunotherapy in the setting of chronic high antigen level and against highly variable pathogens. It is still not clear why T-cells that do not recognise the circulating virus and, therefore, should not be driven to exhaustion by antigen exposure, are still partially exhausted when boosted or primed by our virally-vectored vaccines. Nor is it clear why pre-existing responses, the majority of which also do not target circulating virus, are not boosted by vaccination, or why T-cells in all patients with the virus that is mismatched to the vaccine immunogen sequence at a given epitope do not expand on vaccination. The removal of virus and, therefore, antigen by DAA cure may help to unpick the relative contributions of activation by chronic inflammation and by a specific cognate antigen. The work described here emphasises the need for a particularly broad and cross-reactive T-cell response for the effective control of HCV. These observations are also relevant for vaccine development against other variable pathogens, such as HIV, and for cancer immunotherapy, where mismatch between vaccine immunogen and endogenous antigens will commonly arise.

## 5. Conclusions

In conclusion, we have shown that a highly-potent T-cell vaccine regimen is not able to reconstitute the HCV-specific T-cell pool during chronic HCV disease and that, for variable viruses, it is essential to assess recognition of autologous viral sequence by vaccine-induced T-cells.

## Figures and Tables

**Table 1:** Demographics of HCV infected patients enrolled in HCV003 trial.

Regimen	Patient	Arm	Age (years)	Sex	HCV Genotype	Baseline Viral Load (IU/mL)	HLA-A	HLA-B	HLA-C	HLA-DR	HLA-DQ	RVR	EVR	SVR
14wk IFNa/Rib lead-in pre-vaccination	357	A	58	M	1a	33.7 × 10^6^	02/30	13/62	06/10	04/07	02/08	No	No	No
	362	A	43	M	1a	32,000	01/29	08/44	07/16	01/07/53	02/05	Yes	Yes	No
	364	A	46	F	1a	109,382	03/24	7	07/16	01/103	05	Yes	Yes	No
	366	A	36	F	1a	2.0 × 10^6^	02/33	07/65	07/08	11/103	05/07	Yes	Yes	Yes
2 weeks IFNa/Rib lead-in pre-vaccination	350	B	56	M	1a	1.8 × 10^6^	24/26	35/51	04/15	04/09	08/09	Yes	Yes	No
	351	B	38	M	1b	2.7 × 10^6^	02	36/50	06/10	-	-	Yes	Yes	Yes
	352	B	52	M	1b	9.4 × 10^6^	03/24	07	07	01/15/51	05/06	No	Yes	No
	354	B	51	M	1a	3.2 × 10^6^	01/02	08/62	07/09	52/53	02/08	No	No	No
	356	B	38	M	1b	173,764	11/66	35/41	04/17	13	06	Yes	Yes	w/d
Vaccinated treatment-naïve	355	C	59	M	1a	467,000	02/03	27/65	02/08	52/53	06/07	n/a	n/a	n/a
	358	C	49	F	1a	42,000	03/68	44/60	10/16	07/13/52/53	02/06	n/a	n/a	n/a
	359	C	35	M	1a	237,000	01/66	08/41	07/17	52/53	02	n/a	n/a	n/a
	360	C	51	F	1a	21.7 × 10^6^	02/24	35/44	07/09	04/11/52/53	07	n/a	n/a	n/a

M = male. F = female. RVR= rapid virological response (viral load <50 IU/mL at wk 4 of treatment). EVR = early virological response (viral load undetectable at week 12 of treatment). SVR = sustained virological response, “yes” indicates undetectable viral load 6 months after the end of treatment. n/a = not applicable w/d = withdrawn from study.

**Table 2:** Peptides to which T-cell IFNγ-ELISpot responses were detectable prior to vaccination.

15mer Peptide	Amino Acid Location	Responding Patient	Likely Epitope	Predicted HLA-restriction
AYAAQGYKVLVLNPS	NS3 1243–1252	358	AYAAQGYKVL^	Cw03
LTTGSVVIVGRIILS	NS4A 1677–1686	358	TGSVVIVGR*	A68
TEVDGVRLHRYAPAC & PEFFTEVDGVRLHRY	NS5A 2124–2132	358	EVDGVRLHRY*	A3/B44
LEFWESVFTGLTHID	NS3 1555–1564	358	LEFWESVFTG*	B44/B60
PASSQLDLSGWFVAG	NS5B 2963–2971	351	QLDLSGWFV*	A2

* most likely epitopes––as predicted by epitope prediction program (www.syfpeithi.de; 9 or 10mer with highest prediction score and appropriate HLA based on responding patients HLA-alleles)––are highlighted in bold in 15mer sequences; ^ previously reported epitope [43].

**Table 3:** T-cell responses mapped to individual peptides/pools.

Regimen	Group	Patient	HLA-A	HLA-B	HLA-C	pre-vaccination +ve pools	post vaccination+ve pools	Mapped to peptide (parent pool)
14wk IFNa/Rib lead-in pre-vaccination	A	357	02/30	13/62	06/10	-	G	KLSGLGINAV (G)
		362	01/29	08/44	07/16	-	-	-
		364	03/24	07	07/16	-	F, I, M	-
		366	02/33	07/65	07/08	-	F, G	KLSGLGINAV (G)
2 weeks IFNa/Rib lead-in pre-vaccination	B	350	24/26	35/51	04/15	-	-	-
		351	02	36/50	06/10	M	G, M	KLSGLGINAV (G), PASSQLDLSGWFVAG (M)
		352	03/24	07	07	G, H, I, L	G, H, I	-
		354	01/02	08/62	07/09	-	-	-
Vaccinated treatment-naïve	C	355	02/03	27/65	02/08	-	G, M	KLSGLGINAV (G), SRARPRWFM (M)
		358	03/68	44/60	10/16	F, G, H, I, L, M	F, G, H, I, L, M	AYAAQGYKVLVLNPS (F), LEFWESVFTGLTHID (G), LTTGSVVIVGRIILS (H), TEVDGVRLHRYAPAC (I)
		359	01/66	08/41	07/17	-	F, G, I	HSKKKCDEL (G)
		360	02/24	35/44	07/09	-	G	KLSGLGINAV (G)

T-cell responses to a peptide pool that are only detectable after vaccination are highlighted in red. Responses that were mapped to the epitope or peptide level are shown. If the optimal has been predicted in silico but not tested ex vivo the predicted optimal is shown in bold within the sequence of the 15mer peptide.

**Table 4:** IFNγ ELISpot T-cell responses at baseline, circulating virus sequence vs. vaccine insert. A) HLA-B8 epitope, B) HLA-A2 epitope, C) Epitope of unknown HLA restriction.

(A)	Patient	H	S	K	K	K	C	D	E	L	(B)	Patient	K	L	S	G	L	G	I	N	A	V	(C)	Patient	S	R	A	R	P	R	W	F	M
HLA-B8 + ve pts	359	-	-	-	R	-	-	-	-	-	HLA-A2 +ve pts	357	-	-	V	A	-	-	V	-	-	-		357	-	-	-	-	-	-	-	-	W
	*359pv*	-	-	-	R	-	-	-	-	-		351	-	-	-	A	-	-	L	-	-	-		351	-	-	-	-	-	-	-	-	-
	354	-	-	-	-	-	-	-	-	-		354	-	-	T	A	M	-	-	-	-	-		354	-	H	-	-	-	-	-	-	W
	362	-	-	-	-	-	-	-	-	-		355	-	-	V	A	-	-	V	-	-	-		355	-	-	-	-	-	-	-	-	W
HLA-B8 neg pts	357	-	-	-	-	-	-	-	-	-		*355pv*	-	-	V	A	-	-	V	-	-	-		*355pv*	-	-	-	-	-	-	-	-	W
	350	-	-	-	-	-	-	-	-	-		360	-	-	V	A	-	-	-	-	-	-		360	-	-	-	-	-	-	-	-	W
	351	-	-	-	-	-	-	-	-	-		*360pv*	-	-	V	A	-	-	-	-	-	-		*360pv*	-	-	-	-	-	-	-	-	W
	352	-	-	-	-	-	-	-	-	-		366	-	-	V	S	-	-	-	-	-	-		366	-	H	-	-	-	-	-	-	W
	356	-	-	-	-	-	-	-	-	-	HLA-A2 Neg pts	350	-	-	V	A	-	-	-	-	-	-		350	-	-	-	-	-	-	-	-	W
	355	-	-	-	-	-	-	-	-	-		352	-	-	V	A	-	-	V	-	-	-		352	-	-	-	-	-	-	-	-	W
	*355pv*	-	-	-	-	-	-	-	-	-		356	-	-	-	-	-	-	L	-	-	-		356	-	-	-	-	-	-	-	I	-
	358	-	-	-	-	-	-	-	-	-		358	-	-	V	A	-	-	V	-	-	-		358	-	-	-	-	-	-	-	-	W
	*358 pv*	-	-	-	-	-	-	-	-	-		*358pv*	-	-	V	A	-	-	V	-	-	-		*358pv*	-	-	-	-	-	-	-	-	W
	360	-	-	-	-	-	-	-	-	-		359	-	-	V	A	-	-	V	-	-	-		359	-	-	-	-	-	-	-	-	W
	360pv	-	-	-	-	-	-	-	-	-		359	-	-	V	A	-	-	V	-	-	-		*359pv*	-	-	-	-	-	-	-	-	W
	364	n.d										364	n.d											*364*	n.d								
	*366*	-	-	-	-	-	-	-	-	-		*362*	-	-	V	A	-	-	-	-	-	-		362	-	-	-	-	-	-	-	L	W

Viral sequence was determined at baseline and 2 weeks post-vaccination (pv; untreated patients only). The HCV vaccine immunogen sequence is given in the top line of each table. A dashed line below indicates that an amino acid is identical to that in the vaccine immunogen. The epitopes to which individual patients have made a T-cell response (as measured by ex vivo IFNγ-ELISpot assay) are shown in bold red font.

## Supplementary Materials

The following are available online at www.mdpi.com/2076-393X/4/3/27/s1; Figure S1: Safety data, Figure S2: Phylogenetic tree of HCV whole genome sequences, Figure S3: Anti-vector immunity, Table S1: Study arms, vaccinations given and dose, treatment given.
