# Supplementary material for: Highly-Immunogenic Virally-Vectored T-cell Vaccines Cannot Overcome Subversion of the T-cell Response by HCV during Chronic Infection
Source: Vaccines (Basel). 2016 Aug 2;4(3):27. doi: 10.3390/vaccines4030027 (PMC5041021; doi:10.3390/vaccines4030027)
Supplement: Supplementary file 1 [file vaccines-04-00027-s001.docx]

Supplementary Materials: Highly Immunogenic Virally Vectored T-cell Vaccines Cannot Overcome Subversion of the T-cell Response by HCV during Chronic Infection

Leo Swadling ^1,†^, John Halliday ^1,2,3,†^, Christabel Kelly ^1,2^, Anthony Brown ^1^, Stefania Capone ^4^, M. Azim Ansari ^1^, David Bonsall ^1^, Rachel Richardson ^1^, Felicity Hartnell ^1^, Jane Collier ^2^, Virginia Ammendola ^4^, Mariarosaria Del Sorbo ^4^, Annette Von Delft ^1^, Cinzia Traboni ^4^, Adrian V. S. Hill ^1,5^, Stefano Colloca ^4^, Alfredo Nicosia ^4,6,7^, Riccardo Cortese ^8^, Paul Klenerman ^1,2,5^, Antonella Folgori ^4^ and Eleanor Barnes ^1,2,5,^*

**Figure S1.** Safety data: (**A**) Safety data is shown after ChAd3-NS (top row) and MVA-NS (bottom row) vaccination (combined arms A–C). The percentage of patients with local or systemic adverse reactions (ARs) classified by type and severity (mild, moderate, severe) are shown, and the number of patients receiving each vaccination indicated in parenthesis; (**B**) Kinetics of alanine transaminase (ALT) for vaccinated patients after ChAd3-NS prime (P) vaccination and after MVA-NS boost (B) vaccination. wks = weeks.

**Figure S2.** Phylogenetic tree of HCV whole genome sequences from vaccinated patients and representative sequences for genotype 1a, 1b, 2a and 3a (full length viral sequencing could not be performed for patients 356 and 358). Tested at screening unless otherwise stated.

**Figure S3.** Anti-vector immunity: (**A**) The magnitude of the T-cell response targeting the adenovirus hexon proteins (peptide pool of 15mers overlapping by 11 amino acids covering the hexon of Ad5) was measured by ex vivo IFN-γ ELISpot in HCV infected patients (arms A–C) and healthy volunteers before vaccination, 2, 4, 8 weeks post-ChAd3-NSmut prime vaccination and long-term after prime vaccination (22–36 weeks post ChAd3-NSmut; EOS = end of study). Bars at median, error bars at interquartile range. Kruskal-Wallis multiple comparisons ANOVA with Dunn’s correction between volunteers and patients at each time point. Significant differences are shown; (**B**) The titer of anti-ChAd3 nAbs at baseline and 4 weeks after ChAd3-NSmut vaccination is shown for HCV infected patients (red dots) and healthy volunteers (black stars). Dotted line at nAb titer of 200. Bars at geoMean.

**Table S1.** Study arms, vaccinations given and does, treatment given.

| **Trial** | **Arm** | **n** | **Vaccines + Dose** | **Treatment** |
| --- | --- | --- | --- | --- |
| HCV003 | A | 5 | TW14*: ChAd3-NSmut TW22*: MVA-NSmut | peg-IFNa/Rib (14 weeks lead-in) |
|  | B | 5 | TW2*: ChAd3-NSmut TW10*: MVA-NSmut | peg-IFNa/Rib (2 weeks lead-in) |
|  | C | 4 | TW0: ChAd3-NSmut TW8: MVA-NSmut | - |

Vaccine dose 2.5 × 10^10^ viral particles for Ads and 2 × 10^8^ plaque forming units for MVA; n = number of individuals; * Trial weeks for patients receiving peg-IFNa/Rib refer to weeks after start of treatment (duration of treatment 48 weeks).
